# Octacalcium Phosphate-Laden Hydrogels on 3D-Printed Titanium Biomaterials Improve Corrosion Resistance in Simulated Biological Media

**DOI:** 10.3390/ijms241713135

**Published:** 2023-08-24

**Authors:** Aydin Bordbar-Khiabani, Ilijana Kovrlija, Janis Locs, Dagnija Loca, Michael Gasik

**Affiliations:** 1 Department of Chemical and Metallurgical Engineering, School of Chemical Engineering, Aalto University Foundation, 02150 Espoo, Finland; 2 Rudolfs Cimdins Riga Biomaterials Innovation and Development Centre, Faculty of Materials Science and Applied Chemistry, Institute of General Chemical Engineering, Riga Technical University, Pulka 3, LV-1007 Riga, Latvia; 3 Baltic Biomaterials Centre of Excellence, Headquarters at Riga Technical University, LV-1007 Riga, Latvia

**Keywords:** titanium, implants, octacalcium phosphate, alginate, hydrogel, electrochemical behavior, simulated inflammatory conditions

## Abstract

The inflammatory-associated corrosion of metallic dental and orthopedic implants causes significant complications, which may result in the implant’s failure. The corrosion resistance can be improved with coatings and surface treatments, but at the same time, it might affect the ability of metallic implants to undergo proper osteointegration. In this work, alginate hydrogels with and without octacalcium phosphate (OCP) were made on 3D-printed (patterned) titanium alloys (Ti Group 2 and Ti-Al-V Group 23) to enhance their anticorrosion properties in simulated normal, inflammatory, and severe inflammatory conditions in vitro. Alginate (Alg) and OCP-laden alginate (Alg/OCP) hydrogels were manufactured on the surface of 3D-printed Ti substrates and were characterized with wettability analysis, XRD, and FTIR. The electrochemical characterization of the samples was carried out with open circuit potential, potentiodynamic polarization, and electrochemical impedance spectroscopy (EIS). It was observed that the hydrophilicity of Alg/OCP coatings was higher than that of pure Alg and that OCP phase crystallinity was increased when samples were subjected to simulated biological media. The corrosion resistance of uncoated and coated samples was lower in inflammatory and severe inflammatory environments vs. normal media, but the hydrogel coatings on 3D-printed Ti layers moved the corrosion potential towards more nobler values, reducing the corrosion current density in all simulated solutions. These measurements revealed that OCP particles in the Alg hydrogel matrix noticeably increased the electrical charge transfer resistance at the substrate and coating interface more than with Alg hydrogel alone.

## 1. Introduction

Titanium alloys are the most widely used biomaterials for the manufacturing of dental and orthopedic implants mostly due to their outstanding mechanical properties and desired biocompatibility in the human body environment [1,2,3]. They have reasonable corrosion resistance under normal physiological conditions (pH ≈ 7) as their surfaces are intrinsically covered with dense protective oxide layers [4,5]. However, at inflammatory conditions, reactive oxygen species (ROS), lactic acid, hydroperoxyl radicals (HO_2_), and hypochlorous acid (HOCl, expressed by leukocytes to the extracellular environment) interact with metal surfaces affecting corrosion resistance [6,7,8]. The extracellular medium becomes more acidic (pH ≈ 4–5) when osteoclasts release hydrochloric acid [6], and, subsequently, due to the reactions of neutrophils, microorganisms, and macrophages around an implant affected by the severe inflammation, the environment may become even more oxidative and acidic (pH ≈ 2–3) [9,10].

One approach to address the aforementioned issue is the surface modification of the implants, which is a reasonably powerful strategy to enhance corrosion resistance [11,12]. For example, hydrogels have been extensively proven as excellent surface coatings for Ti implants due to their chemical stability, viscoelasticity, hydrophilicity, and biocompatibility [13,14]. Among them, sodium alginate (Alg) biopolymer coatings with high bioactivity, good biocompatibility, and cost-effectiveness has been broadly studied [15,16]. However, pure alginate exhibited poor mechanical properties and low activity towards the cells [17,18], and in addition, the adhesion of hydrogel and biopolymer coatings on flat metallic implants is reported to be weak [19]. Therefore, an intermediate surface modification using 3D techniques is usually needed for the better adhesion of these layers to metallic implants [20,21]. Additive manufacturing techniques can be an effective method to 3D print specific patterns on the implant surface [22]. In recent years, 3D-printed Ti biomaterials also showed great potential in promoting bioactivity and osseointegration on their surface, and such techniques have been employed to boost the corrosion resistance of Ti biomaterials in biological media [23]. Implants with rough or patterned surfaces may enhance the interlock between the hydrogel and the substrate [13,19], and as known, the implant surface roughness plays a crucial role in cellular and simultaneous bacterial colonization [24,25].

In general, there is a need for a suitable surface modification and/or a coating that will provide enhanced biocompatibility and osteointegration, being well adhered, and might offer potential for the ameliorated antibacterial properties or delivery of drugs or ions [26,27]. For this reason, titanium implants covered with a polymer can also be modified with bioactive inorganic materials such as calcium phosphates (CaPs) to improve the implant’s biological function [28,29,30], while the environmental conditions on the implant surface with the presence of PO_4_^3−^, Ca^2+^ ions, and biomolecules in a wet environment are similar to those that are physiologically required for osteogenesis and osteoblast activities [31,32].

Octacalcium phosphate (OCP) has consistently ranked as one of the most promising calcium phosphates for the regeneration of bone tissue [26,33]. In vitro and in vivo studies demonstrated that OCP could be used as a material that improves the osteogenic condition of non-cell-interactive polymeric scaffolds, such as Alg [34,35,36]. However, synthesizing OCP depends on several factors (e.g., pH, temperature, precursors, duration, etc.), which can be the cause of phase impurities such as a mix of two or more CaPs or can lead to a premature transition into non-active apatite [37,38]. Due to its unique structure that has high similarities to hydroxyapatite (HAp) and the presence of a water layer, OCP has great potential for incorporating different drugs or ions, ensuring local drug release, and providing the antibacterial effect. On the other hand, its sensitive structure and readiness to convert to a more stable hydroxyapatite, when in contact with biological fluids, is what limits the use of OCP [39].

A titanium surface with hydrogels becomes hydrophilic and, hence, more capable of accumulating corrosive ions at the gel and substrate interface, changing the electrochemical corrosion behavior. Recent studies have shown that the presence of a layer of hydrogel on magnesium and nitinol implants significantly increases their corrosion resistance [40,41]. When a mixture of ACP and chitosan hydrogels has been used on the Ti, it exhibited a high corrosion resistance [42], but when porous calcium carbonate (CaCO_3_) was mixed with alginate, the corrosion resistance was decreased [43]. Authors are not aware of systematic quantitative studies on the corrosion resistance of OCP-laden Alg hydrogels, especially when they are used as coatings on 3D-patterned titanium alloys. Therefore, the aim of this work is to study and compare the corrosion behavior of 3D-patterned Ti surfaces coated with Alg and a 3 wt% Alg:crystalline OCP phase (70:30 wt% inorganic:organic ratio) upon immersion in the media for simulated normal, inflammatory, and severe inflammatory conditions.

## 2. Results and Discussion

### 2.1. Coating Morphology and Wettability

**Figure 1:**
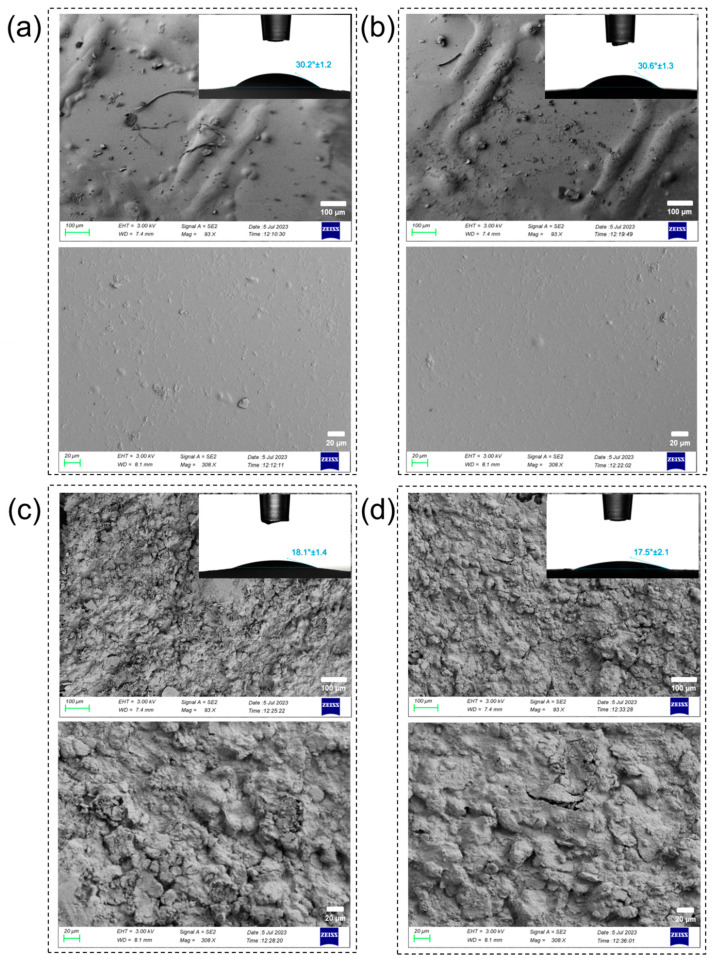
FESEM micrographs of Alg coating on 3D-printed (**a**) Ti Gr2 and (**b**) Ti Gr23 and Alg/OCP coatings on (**c**) Ti Gr2 and (**d**) Ti Gr23. The PBS solution droplet contact angle for hydrogel coatings on 3D-printed substrates are shown in insert images.

The morphology of the Alg and Alg/OCP coatings on the 3D-printed Ti Gr2 and Gr23 substrates are shown in Figure 1, in two different magnifications. It was found that different 3D-printed Ti substrates had no noticeable effect on the morphologies of the hydrogel coatings, which were qualitatively quite similar. Low-magnification images also demonstrated uniform distribution and the complete covering of the square patterns by both hydrogels.

The inserted images in Figure 1 show the effect of hydrogel coatings on the surface wettability of patterned Ti groups. It can be seen that the contact angles on the hydrogel-coated samples were lower than those on the patterned Ti group [23], which indicates that these hydrogel coatings improved the wettability. The main reason for this might be the presence of hydrophilic functional groups such as carboxyl and hydroxyl in Alg [44]. It was also found that the introduction of OCP into Alg coatings reduced the contact angles. This might be mainly attributed to the increase in surface free energy due to the presence of OCP. Moreover, the presence of OCP particles in the form of agglomerates further increases the wettability of composite coatings. The space between the agglomerated OCP particles is larger than that of the smaller particles in the accumulation process because they cause a larger equivalent capillary radius, thus increasing the wetting rate [45].

### 2.2. Physicochemical Characterization of Alg/OCP Coatings

**Figure 2:**
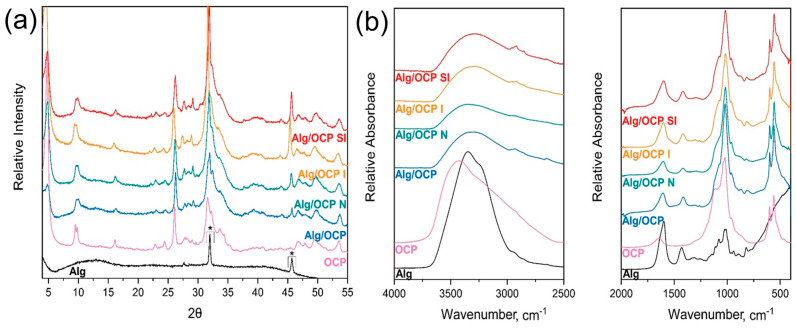
Physicochemical characterization of alginate, OCP, and the coating prior to the immersion (Alg/OCP) and after 1 h immersion in simulated normal (Alg/OCP N), inflammatory (Alg/OCP I), and severe inflammatory (Alg/OCP SI) conditions. (**a**) X-ray diffractograms; (**b**) FTIR spectra.

The OCP powder, synthesized from low-temperature α-TCP, used in the present study was physicochemically well characterized in our previous report [37]. A low-angle (100) maximum at 2θ = 4.7 degrees and a doublet at 9.4 and 9.7 (200 and 010) 2θ degrees, which are the characteristic X-ray diffraction fingerprints of OCP, were observed (Figure 2a). The FTIR spectrum showed the vibrations of HPO_4_^2−^ at 917, 875, 1007, and 1295 cm^−1^, which differentiate the OCP from stoichiometric hydroxyapatite (Figure 2b) [46,47]. The specific amorphous broad halo that was seen in the XRD pattern was specific to sodium-alginate biopolymer, and it aligned with the results from the FTIR spectra that showed a broad band centered at approximately 3500 cm^−^^1^ from the stretching of hydroxyl groups. The crystalline peaks correspond to calcium chloride, which was present in the crosslinking process (Figure 2a—star symbol). Once the composite was analyzed by FTIR, a set of several vibrations in the range of 1100–1000 cm^−^^1^, connected to the glycoside bonds in the polysaccharide (C-O-C stretching), was seen [48].

Due to its specific structure that comprises a water channel in between apatite layers, OCP is unable to be sintered or morphed in any way with the help of high temperatures [39]. On the other hand, combining it with polymers that require a more complex crosslinking process (e.g., a pH change or the addition of more components) could potentially cause the OCP phase to transform to hydroxyapatite. A combination of 3 wt% Alg solution and OCP, in the used ratio, was intended to preserve the crystalline form of OCP and provide a strong protective coating on the titanium alloys. In both XRD and FTIR data, the characteristic maxima of OCP were maintained after the Alg/OCPcoating preparation, confirming that the Alg did not cause subsequent modifications in the crystal structure of OCP. The decrease in intensity and the broadening of the 25–35° 2θ area indicate the decrease in the crystallinity in Alg/OCP. However, once the coatings have been immersed into the respective solutions, CaP crystallinity intensifies again, and the OCP phase remains stable. This could potentially be because of the presence of different salts and ions in the media (i.e., BSA) that are beneficial towards the OCP as they slow down the hydrolysis via the absorption onto the OCP surface [49]. In FTIR spectra, the absorbance bands at 1620 and 1416 cm^−^^1^, which were seen in pure Alg and in all tested coatings, corresponded to the antisymmetric and symmetric COO stretching vibration of the salified carboxyl group of alginic acid [50,51,52]. As it was seen also in the XRD patterns, the broadening of the ν_3_PO_4_ region (900–1200 cm^−^^1^) is consistent with the reduction in OCP crystallinity when combined with Alg.

### 2.3. Electrochemical Studies

#### 2.3.1. Open Circuit Potential

**Figure 3:**
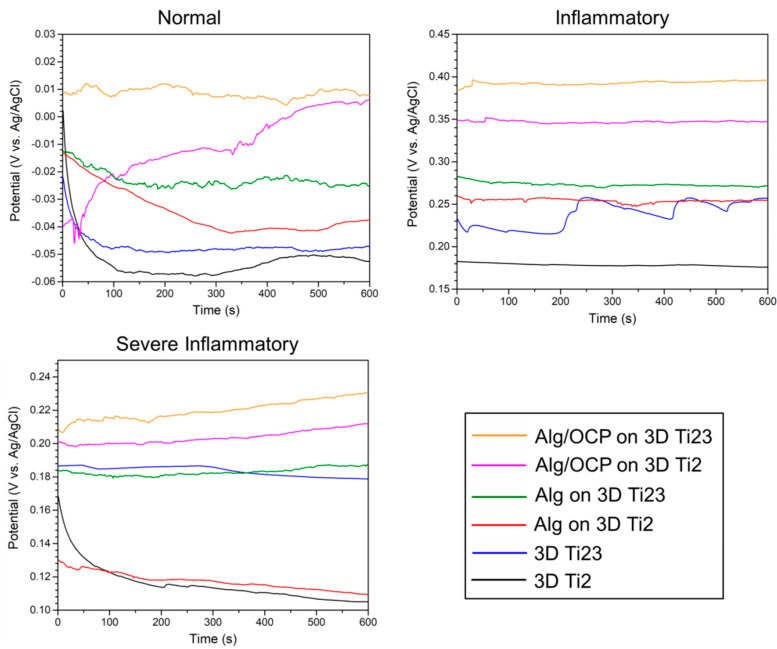
Open circuit potential monitoring of the prepared Alg and Alg/OCP coatings on 3D-printed Ti Gr2 and Ti Gr23 layers exposed in simulatednormal, inflammatory, and severe inflammatory conditions for 1 h.

The changes in open circuit potential values as a function of time for the bare 3D-printed Ti groups and those coated with Alg and Alg/OCP hydrogels coatings in simulated media at 37 °C are shown in Figure 3. During electrochemical oxidation, the open circuit potential represents the thermodynamic tendency of the material. Both the Alg and Alg/OCP coatings on the Ti Gr2 and Ti Gr23 substrates showed higher values of potential at the end of 600 s in all simulated media than the bare samples, confirming the favorable effect of the coatings [53]. However, a small difference in the final recorded potential in the Alg-coated Ti Gr2 and Ti Gr23 samples in the severe inflammatory solution can be seen, which could be related to its low stability in low pH solutions. The obtained results revealed that the Alg/OCP coatings exhibited a higher potential than Alg on both substrates, indicating the positive role of OCP particles in the electrochemical performance of Alg.

By comparing Alg- and Alg/OCP-coated samples, it has been determined that the distribution of OCP particles in the Alg matrix creates a greater open circuit potential difference in the inflammatory medium than in the severe inflammatory medium, suggesting that these particles are capable of effective action in pH 5 and in the absence of BSA and lactate. Another noticeable variation is about the open circuit potential of hydrogel-coated Ti Gr2 in a normal medium. The potential of Alg-coated Ti Gr2 (red line) initially decreased over time during the 300 s, then increased slightly, and stabilized at −330 mV_Ag/AgCl_. This indicates the dissolution of the developed oxide film during the 300 s and its subsequent growth at 600 s [54]. The Alg/OCP-coated Ti Gr2 (purple line) showed different behavior. The open circuit potential fluctuated during the first 50 s and then increased to positive values continuously. It shows the dissolution and growth of oxide film simultaneously in the first 50 s of immersion and then the subsequent growth by the end of the test. The results also showed that regardless of the type of hydrogel on the 3D-printed Ti substrates, Ti Gr23 has a more electropositive potential than Ti Gr2, which is likely to be due to the formation of dense composite oxide layers on Ti Gr23 [55]. During simulated inflammatory and severe inflammatory conditions, open circuit potential values moved toward electropositive potentials. The decomposition of H_2_O_2_ into H_2_ and O_2_ may explain this phenomenon, adding to the sluggish oxygen reduction reaction by acting as an additional cathodic reaction [56]. The presence of BSA and CLH in the inflammatory solution resulted in a decrease in open circuit potential values in severe inflammatory conditions. Still, potential values remained high and more positive than those of normal conditions.

#### 2.3.2. Potentiodynamic Polarization

**Figure 4:**
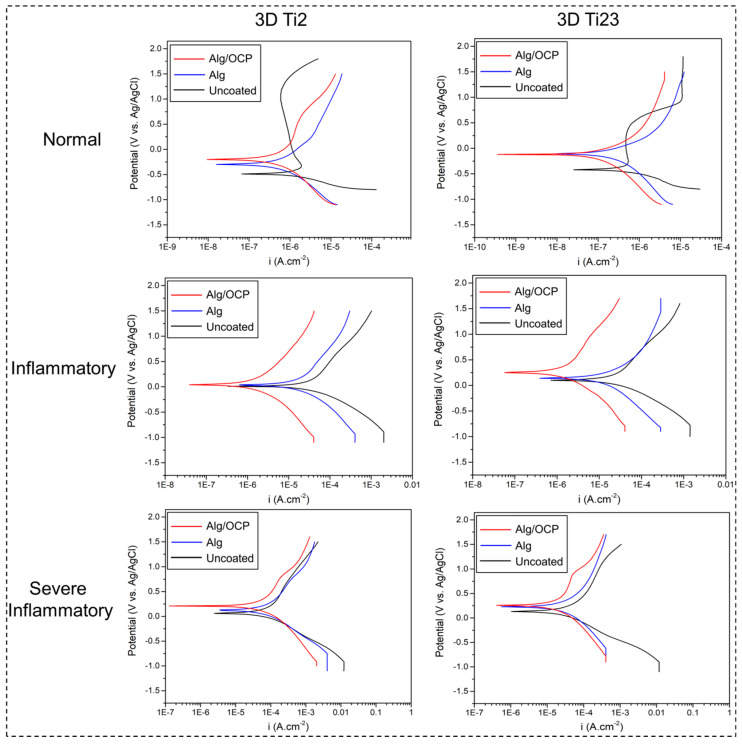
Potentiodynamic polarization curves of the prepared Alg and Alg/OCP coatings on 3D-printed Ti Gr2 and Ti Gr23 layers exposed in simulated normal, inflammatory, and severe inflammatory conditions for 1 h.

The potentiodynamic polarization curves of hydrogel coatings on 3D-printed Ti samples are presented in Figure 4. The obtained parameters from the Tafel extrapolation method, including corrosion potentials (E_corr_), corrosion current densities (I_corr_), and Tafel slopes of anodic (β_a_) and cathodic (β_c_) branches, as well as calculated polarization resistance using the Stern–Geary equation [57], are listed in Supplementary Table S1. The obtained results indicated that under inflammatory and severe inflammatory conditions, samples are incapable of exhibiting passive behavior due to the presence of H_2_O_2_ in their media [58]. It is noteworthy that anodic branches do not exhibit Tafel behavior under normal conditions, thus rendering the Stern–Geary analysis invalid [59]. Generally, excellent anticorrosion properties are largely dependent on an electropositive E_corr_, low I_corr_, and high R_p_. It can be seen from Table S1 that the i_corr_ of the hydrogel-coated Ti Gr23 is lower than that of the coated Gr2 in all conditions, which confirms the fact that the corrosion resistance of the substrate affects the electrochemical performance of the coatings. By comparing the obtained parameters for uncoated and coated samples, it was found that a layer of hydrogel improves the corrosion resistance of 3D-printed Ti Gr2 and Gr23 in all media.

Enhanced corrosion resistance for coated surfaces may be related to the hydrogel layers’ ability to protect the substrate both physically and chemically [60,61]. As seen in Table S1, Alg/OCP-coated Ti groups have nobler E_corr_, lower I_corr_, and higher R_p_ than Alg-coated samples in all simulated biological media. This is attributed to the fact that OCP inorganic particles with a low tendency for electrochemical reactions functioned as corrosion-inhibiting barriers and provided effective physical separation between the substrate and corrosive medium. The drastic decrease in the i_corr_ of hydrogel-coated samples immersed in the presence of the H_2_O_2_, BSA, and CLH compounds confirms, once again, the increase in the reactivity of 3D-printed Ti substrates under these conditions [7,23]. Furthermore, it also confirms the decrease in corrosion resistance via the penetration of corrosive ions through hydrogel to the substrate.

#### 2.3.3. Electrochemical Impedance Spectroscopy

**Figure 5:**
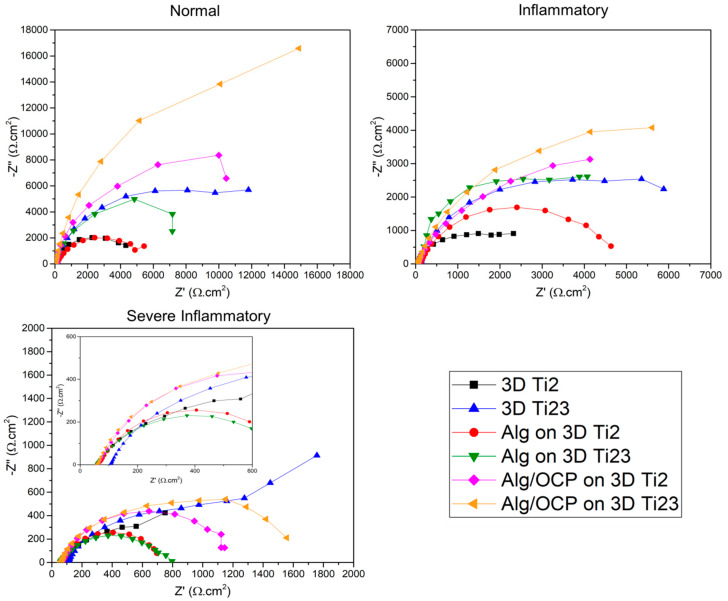
EIS plots of the prepared Alg and Alg/OCP coatings on 3D-printed Ti Gr2 and Ti Gr23 layers exposed in simulated normal, inflammatory, and severe inflammatory conditions for 1 h: Nyquist plots. Nyquist plots for the severe inflammatory condition have been enlarged for better display.

**Figure 6:**
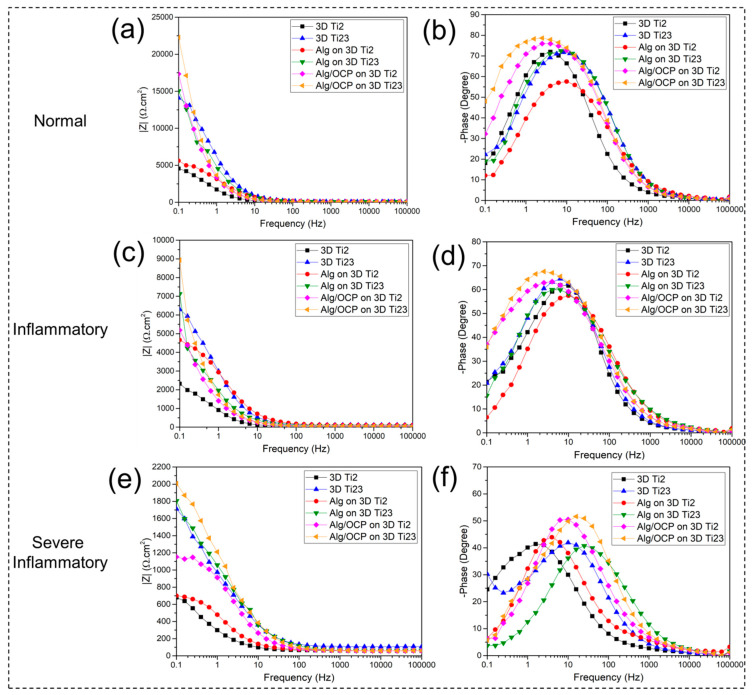
EIS plots of the prepared Alg and Alg/OCP coatings on 3D-printed Ti Gr2 and Ti Gr23 layers exposed in simulated (**a**) normal, (**b**) inflammatory, and (**c**) severe inflammatory conditions for 1 h: (**a**–**c**) Bode modulus and (**d**–**f**) Bode phase plots.

The Nyquist and the Bode amplitude/phase plots of the impedance data for bare and hydrogel-coated 3D-printed Ti groups in simulated media are presented in Figure 5 and Figure 6, respectively. Figure 5 shows a decreasing trend in capacitive loop diameters of Nyquist plots for inflammatory and severe inflammatory conditions, suggesting a subsequent decrease in corrosion resistance with H_2_O_2_, BSA, and LCH added to PBS. The Bode amplitude plot also confirmed the same trend in the low-frequency range of the impedance modulus (Figure 6a–e). Under severe inflammatory conditions, phase angles of about 40° and 50° in Figure 6f suggest that there is a high corrosion rate for all samples. The equivalent circuit according to the fitted results of the plots is shown in Supplementary Figure S1 to quantitatively investigate the impedance values of the coated samples. Equivalent circuits for uncoated 3D-printed Ti groups have been shown in previous work [23]. In the proposed circuit in this study, R_s_ represents the solution’s resistance between the R.E. and W.E. In order to express the capacitive properties of coating surfaces due to the nonhomogeneous nature of the coatings, the constant phase element (CPE) is applied [62]:

(1) $$Z_{CPE}=\frac{1}{Q{\left(j\omega \right)}^{n}}$$

where j^2^ = −1, ω is an angular frequency (rad.s^−^^1^), and n = 0…1 is the power coefficient (with n = 1, CPE behaves as a pure capacitance, and for n = 0, it behaves as a pure resistance). In the circuit, an electrical double layer’s capacitance at the interface between a substrate and a solution is represented by CPE_dl_, and its resistance to charge transfer is represented by R_ct_. The R_c_ and CPE_c_ describe the resistance and capacitance of hydrogel coatings. Supplementary Table S2 summarizes the parameters that correspond to the fitted results. A hydrogel coatings’ barrier property is evaluated by R_ct_ and R_c_ values. Greater R_ct_ implies a more difficult charge transfer and less dissolution of a substrate.

The obtained results showed that the R_ct_ values of hydrogel-coated samples were significantly higher than those of the 3D-printed Ti groups, indicating that the hydrogel coating creates a strong barrier that prevents corrosive ions’ penetration to the substrate [63]. The hydrogel coatings had proper adhesion to substrates before and after electrochemical studies. For example, they were strong enough to withstand handling under flowing water jets. The R_ct_ of Alg/OCP-coated samples also increased as OCP particles were incorporated into Alg, suggesting that the OCP particles improved the barrier effect of coatings against electron transfer on the 3D-printed Ti surface. Furthermore, it was found that Alg hydrogel’s R_c_ was further enhanced by the addition of OCP, suggesting that the OCP particles might strengthen the crosslinking degree of the Alg hydrogel coating and the interfacial bonding force between the coating and the substrate, resulting in the increase in density of hydrogel coatings, and thus, the Alg/OCP-coated samples exhibited higher R_c_ than samples coated with pure Alg.

The decrease in the R_c_ values of coated samples in inflammatory and severe inflammatory conditions compared with normal conditions can be explained by the hydrogels becoming unstable following their dissolution by HCl and H_2_O_2_, which leads to a decrease in R_ct_ or an increase in substrate reactivity in these media. Usually, reduction and oxidation can cause the decomposition of disulfide bonds due to the presence of reductants, thus resulting in redox-responsive hydrogels [64]. In addition, by comparing R_c_ values in these conditions, it can be seen that hydrogel coatings in the inflammatory solution provide better protection of the substrate. In order to avoid the aforementioned issues, it is of paramount value to choose a polymer that can withstand the inflammatory and severe inflammatory conditions. In the literature, it has been shown that alginate exhibits relatively good resistance to degradation in moderate concentrations of H_2_O_2_ at 25 °C [65]. It has also been reported that Alg has a much lower degradation and polymerization rate in pH 5–9 compared to highly acidic solutions (pH < 3) [66]. In Alg, ionotropic gelation is induced by bivalent (i.e., Ca^2+^) or polyvalent cations that ionically crosslink carboxylate groups in the uronate blocks, leading to a gel with low solubility at low pH [67]. Therefore, the reduction in corrosion resistance of Alg-based hydrogel coatings in a severe inflammatory solution can be related to the presence of lactate and protein in this medium. Up to now, the effect of lactic acid on alginate has not been completely investigated. However, several models have been proposed regarding the interaction of proteins with Alg. In the most common of these models, the charged BSA in an acidic solution makes a connection between the positively charged parts of the protein and the carboxyl groups of Alg via electrostatic force [68]. On the other hand, the binding of corrosive ions to the protein branches and their pulling towards the substrate and increasing the corrosion rate have also been proven [69]. Thereby, proteins that act as connectors between corrosive ions and hydrogel coatings can be a good justification for reducing corrosion resistance in a severe inflammatory medium. An accurate elucidation of the effect of protein and organic acid on the structural change and corrosion behavior of the Alg-based composite should be suggested in future studies.

## 3. Materials and Method

### 3.1. Preparation of 3D-Printed Ti Alloys

The supplied Ti Gr2 (99.6% Ti) and Ti Gr23 alloy (Al 5.5…6.76%, V 3.5…4.5% wt., Ti—balance) plates by Goodfellow Cambridge Ltd. (Huntingdon, England) were cut into disks with 100 mm diameter and used as substrates. The Ti Gr2 (99.3% Ti), and Ti Gr23 (Al 5.4%, V 3.8%, Fe 0.2%, C 0.009% wt., balance Ti) powders (Merck, Germany) were used for laser-driven powder bed fusion (L-PBF) process. A 3D selective L-PBF printer (Mysint100, Sisma S.p.A., Vicenza, Italy) was used to overprint Ti Gr2 and Ti Gr23 layers in a square-patterned shape on their flat counterparts at Btech Innovation Ltd., Istanbul, Türkiye. After the 3D-printng process, the built parts were cut off into 15 mm diameter small disks (Supplementary Figure S2) with wire electrical discharge machining and washed with water and acetone. A scanning electron microscope (SEM, Hitachi TM-4000Plus-RAMI, Japan) was used to obtain images of 3D-printed TiGr2 and Ti Gr23 (Supplementary Figure S3a, respectively). A detailed description of the fabrication conditions and related parameters can be found in a previous study [23].

### 3.2. Synthesis of Octacalcium Phosphate

OCP was synthesized according to a previously published protocol [37]. Briefly, 1 g of in-house-synthesized α-tricalcium phosphate (α-TCP, 99% α-TCP) was mixed with 500 mL of 0.0016 M orthophosphoric acid (H_3_PO_4_, 75%, Latvijas Ķīmija) at room temperature and stirred (350 rpm) for 72 h with a monitored pH throughout the entire synthesis. The final product was washed with deionized water and left to freely dry overnight (37 °C). The syntheses were replicated more than three times to claim the repetitive formation of OCP.

### 3.3. Synthesis of Octacalcium-Phosphate-Laden Alginate Coatings

**Figure 7:**
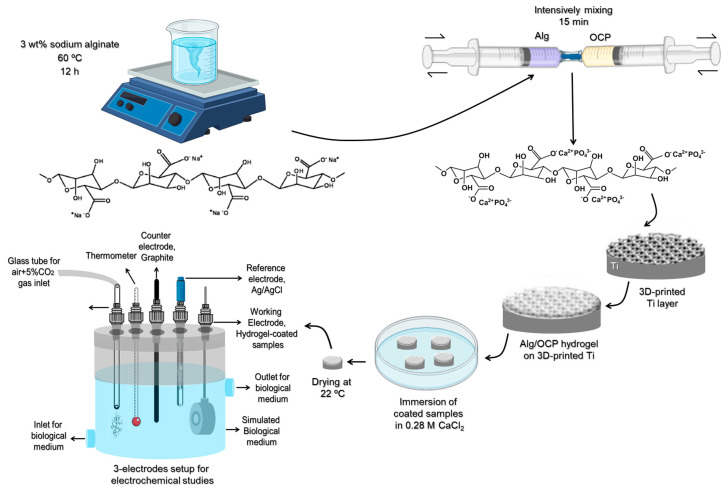
Schematic illustration of preparing Alg/OCP hydrogel coatings on 3D-printed Ti samples for electrochemical studies.

Sodium alginate (9005-38-3, Sigma Aldrich) was dissolved in the deionized water at 60 °C overnight to the final solution concentration of 3 wt% Alg. The Alg/OCP composites were prepared by intensively mixing the solid and liquid phases for 15 min, until a homogenous mass was formed. Up to 10 mg of Alg and Alg/OCP hydrogels were uniformly distributed over the 3D-printed Ti alloys, followed by immersion in 0.28 M CaCl_2_ (A1308701911, Merck) for 15 min to crosslink (Supplementary Figure S4). Compared to Alg hydrogel coatings, which are transparent, the resulting Alg/OCP hydrogel displayed a white, opaque color. The prepared coatings were further used for electrochemical measurements, as shown in Figure 7.

### 3.4. Physicochemical Characterization of the Alg/OCP Coatings

The presence of the OCP phase after the OCP synthesis and in Alg/OCP coatings was examined using X-ray powder diffractometry (XRD). XRD was performed with a PANalytical Aeris diffractometer (The Netherlands) with the following parameters: 40 kV, 15 mA, and a step size of 0.0435° from 3° to 60° (2θ degrees) with a time per step of 299.575 s. The International Centre for Diffraction Data PDF-2 (ICDD) database was used to corroborate the phase presence. For crystalline phase identification, ICDD#026-1056 has been used. Fourier-transform infrared spectroscopy (FTIR, Thermo Scientific Nicolet™ iSTM50 (Waltham, MA, USA)), used in Attenuated Total Reflectance (ATR) mode, was engaged to characterize functional groups of OCP and Alg/OCP at the molecular level with the spectra in the range of 4000–400 cm^−1^ (number of scans was 64 at a resolution of 4 cm^−1^) and processed with the OMNIC software (Thermo Scientific). The Alg/OCP coatings have been tested prior to and after immersion in the relevant testing media.

### 3.5. Coating Characterization

The morphology of the Alg and Alg/OCP hydrogel coatings on 3D-printed Ti Gr2 and Gr23 was evaluated using field-emission scanning electron microscopy (FESEM, Zeiss Sigma VP, Germany). Samples were sputter-coated with Au/Pt prior to the FESEM observations. The FESEM observations were performed at different magnifications in secondary electron (SE) mode with the accelerating voltage of 3 kV. The wettability of hydrogel coatings was assessed using a Theta Flex optical tensiometer (Biolin Scientific, Finland) in sessile drop mode by measuring the contact angles of the 5 μL phosphate-buffered saline (PBS) solution’s droplet. All wettability measurements were conducted under the same conditions of humidity (~60% RH) and temperature (24 °C).

### 3.6. Electrochemical Measurements

Electrochemical tests were performed with a potentiostat (IviumStat.h standard, The Netherlands) in a 3-electrode setup with a Ag/AgCl electrode as a reference electrode (R.E.), a graphite rod as the counter electrode, and hydrogel-coated samples with an exposed area of 1 cm^2^ in the Teflon holder as a working electrode (W.E.) (see Figure 7). The electrochemical response of the samples was evaluated in three different simulated biological media (see Table 1) at 37 ± 0.5 °C under slight anaerobic conditions by an aerating mixture of air + 5% CO_2_ using solutions. The normal medium was made by dissolving PBS tablets (Sigma-Aldrich, USA) in l L of distilled water. For the simulation of the inflammatory conditions, hydrochloric acid (HCl; 37%, Merck, Germany) and H_2_O_2_ (30%, Merck, Germany) were added to the normal medium. For mimicking the severe inflammatory conditions, bovine serum albumin (BSA, Sigma-Aldrich, Germany) and calcium L-lactate hydrate (CLH, Sigma-Aldrich, Germany) were all added to the above inflammatory medium.

The conductivity and pH values of the simulated solutions were measured using a Mettler Toledo Inlab 730 probe and a Metrohm 691 pH meter, respectively. Considering H_2_O_2_ instability under light, all of the solutions were stored in dark bottles until tests.

The electrochemical response was analyzed based on open circuit potential, potentiodynamic polarization, and electrochemical impedance spectroscopy (EIS) tests. Before measurements, the samples were soaked in each medium for 1 h. The samples’ open circuit potential was monitored during 600 s. Potentiodynamic polarization measurement was conducted under a constant sweep rate of 1 mV.s^−1^ from −1.5 to 1.5 V. The EIS test was performed under an AC voltage of 10 mV in amplitude around open circuit potential in the frequency range of 100 kHz–0.010 Hz. The EIS plots were examined and fitted by the ZView software (version 3.1, Scribner Associates) to achieve a proper equivalent circuit. Chi-squared (χ^2^) values in the order of 10^−^^3^ confirmed the quality of data fitting. All tests were conducted in triplicate to ensure reproducibility and reliability.

## 4. Conclusions

The inflammation process in the tissues surrounding Ti-based biomaterials can lead to increased acidity with the release of H_2_O_2_ and acids, weakening the corrosion resistance of these implanted devices. The results of this study showed that bare 3D-printed Ti substrates undergo electrochemical corrosion via chemical reactions between the Ti and the corrosive ions of inflammatory and severe inflammatory solutions and that hydrogel coatings might indeed act as a protective film for preventing the chloride and lactate ions in the simulated biological media from corroding the substrate, thereby improving the corrosion resistance of the Ti alloy. According to polarization and EIS study data, the introduction of OCP particles in the Alg hydrogel matrix decreases the corrosion current i_corr_, leading to a notable improvement in the charge transfer resistance increase. The excellent corrosion protection of composite coatings eventually arises from blocking the transport pathway of corrosive ions with OCP particles in the hydrogel. These findings may open new opportunities for the surface engineering of Ti implants with the combination of additive manufacturing and modified hydrogel coatings, providing a potential method for the protection of metallic biomaterials during inflammatory processes.

## Figures and Tables

**Table 1:** Simulated biological media with their respective compositions, pH, and conductivity at 37 °C.

Simulated Biological Media	Reagents and Amounts					pH	Conductivity (mS.cm^−1^)
	PBS	H_2_O_2_	HCl	BSA	CLH		
Normal (N)	Five standard tablets in 1 L of deionized water	-	-	-	-	7.4 ± 0.1	15.7 ± 0.5
Inflammatory (I)		150 mM	50 mM	-	-	5.2 ± 0.1	19.4 ± 0.3
Severe inflammatory (SI)				10 g.L^−1^	10 g.L^−1^	3.0 ± 0.2	16.1 ± 0.1

## Data Availability

The data presented in this study are available on request from the corresponding author. The data are not publicly available due to the connected study.

## Supplementary Materials

The following supporting information can be downloaded at https://www.mdpi.com/article/10.3390/ijms241713135/s1.
