# Supplementary material for: Octacalcium Phosphate-Laden Hydrogels on 3D-Printed Titanium Biomaterials Improve Corrosion Resistance in Simulated Biological Media
Source: Int J Mol Sci. 2023 Aug 24;24(17):13135. doi: 10.3390/ijms241713135 (PMC10487990; doi:10.3390/ijms241713135)
Supplement: Supplementary file 1 [file ijms-24-13135-s001.zip › ijms-2544952-supplementary.pdf]

<u>Suplammetry information</u>

Octacalcium Phosphate-Laden Hydrogels on 3D-Printed Titanium Biomaterials Improve Corrosion Resistance in Simulated Biological Media

Aydin Bordbar Khiabani[1,*], Ilijana Kovrlija[2,3], Janis Locs[2,3], Dagnija Loca[2,3], Michael Gasik[1]

[1]Department of Chemical and Metallurgical Engineering, School of Chemical Engineering, Aalto University Foundation, 02150 Espoo, Finland
[2]Rudolfs Cimdins Riga Biomaterials Innovations and Development Centre of RTU, Institute of General 7 Chemical Engineering, Faculty of Materials Science and Applied Chemistry, Riga Technical University, 8 Riga, Latvia
[3]Baltic Biomaterials Centre of Excellence, Headquarters at Riga Technical University, Riga, Latvia

*Correspondence: aydin.bordbarkhiabani@aalto.fi

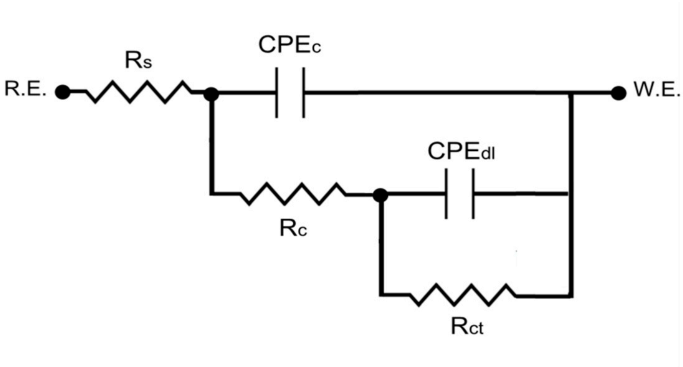

Figure S1. Equivalent circuit model for hydrogel coatings on 3D-printed Ti layers.

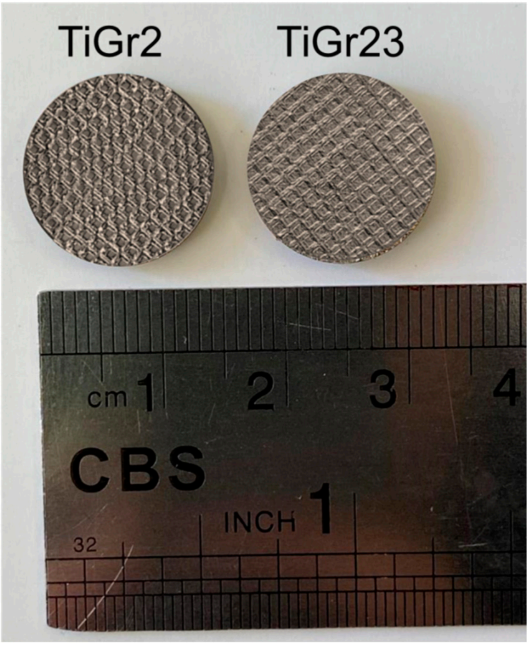

Figure S2. Camera image of the different 3D-printed Ti layers on their counterpart's substrates.

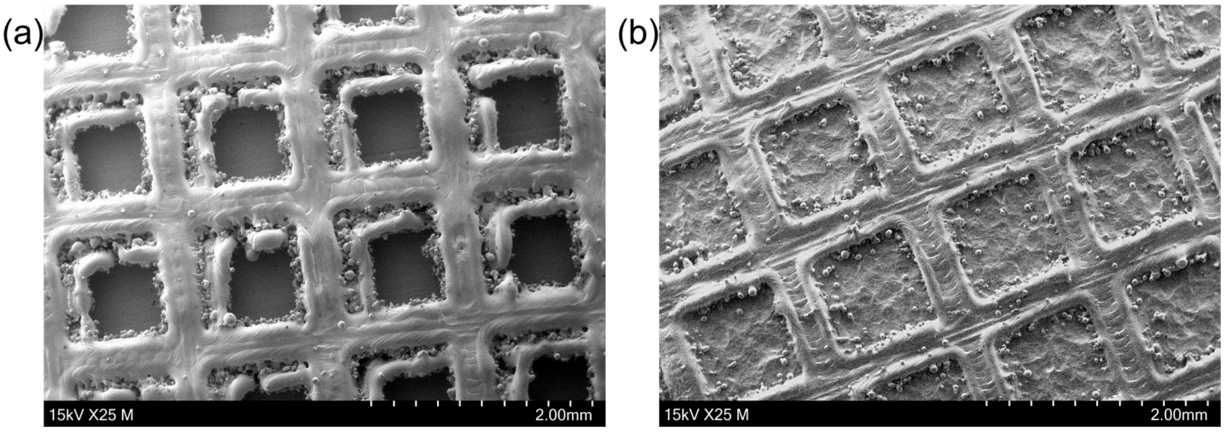

Figure S3. SEM images of the 3D-printed (a) TiGr2, and (b) TiGr23 layers on their counterpart's substrates.

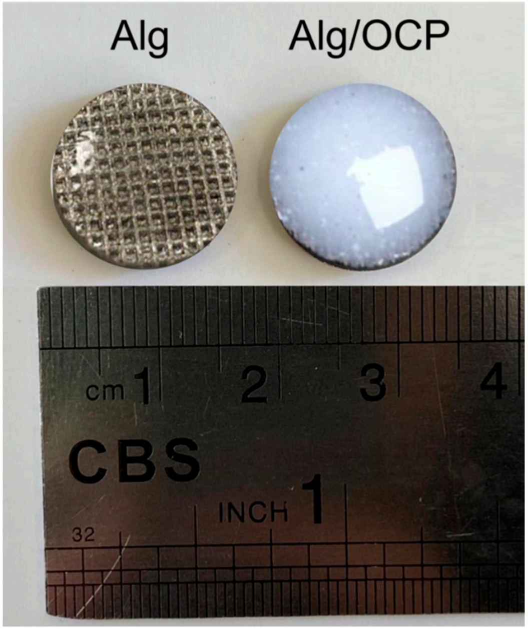

Figure S4. Camera image of the different hydrogel coatings on 3D-printed Ti Gr23 layers.

Supplementary Table S1. Potentiodynamic polarization parameters of the hydrogel coatings on 3D-printed Ti Gr2 and Ti Gr23 substrates (shaded cells) in comparison with uncoated substrates from [14] in different simulated conditions.

Simulated conditions	Specimens	E_{corr} (V vs. Ag/AgCl)	β_a ($V \cdot dec^{-1}$)	$-\beta_c$ ($V \cdot dec^{-1}$)	I_{corr} ($\mu A \cdot cm^{-2}$)	R_p ($k\Omega \cdot cm^2$)
Normal	3D Ti2 [14]	-0.425±0.160	-	0.450±0.140	0.54±0.05	-
	3D Ti23 [14]	-0.537±0.185	-	0.374±0.116	0.23±0.04	-
	Alg on 3D Ti2	-0.222±0.151	-	0.562±0.209	0.37±0.08	-
	Alg/OCP on 3D Ti2	-0.210±0.115	-	0.421±0.105	0.09±0.02	-
	Alg on 3D Ti23	-0.220±0.127	-	0.389±0.163	0.12±0.01	-
	Alg/OCP on 3D Ti23	-0.214±0.158	-	0.412±0.50	0.02±0.008	-
Inflammatory	3D Ti2 [14]	0.231±0.033	0.848±0.144	0.429±0.162	35.12±1.39	3.48±0.47
	3D Ti23 [14]	0.248±0.047	0.718±0.100	0.568±0.101	20.37±0.76	5.95±0.41
	Alg on 3D Ti2	0.233±0.111	0.715±0.079	0.506±0.109	28.08±0.29	4.52±0.15
	Alg/OCP on 3D Ti2	0.251±0.124	0.692±0.263	0.551±0.177	3.85±0.52	34.19±0.30
	Alg on 3D Ti23	0.276±0.33	0.631±0.091	0.574±0.123	17.49±1.01	6.53±0.42
	Alg/OCP on 3D Ti23	0.282±0.082	0.782±0.205	0.473±0.186	0.95±0.11	133.12±1.44
Severe inflammatory	3D Ti2 [14]	0.120±0.066	0.787±0.185	0.422±0.129	67.18±1.80	2.96±0.16
	3D Ti23[14]	0.143±0.011	0.817±0.118	0.532±0.137	45.05±2.69	3.69±0.13
	Alg on 3D Ti2	0.126±0.005	0.809±0.152	0.462±0.181	55.75±0.40	2.26±0.36
	Alg/OCP on 3D Ti2	0.149±0.143	0.794±0.144	0.577±0.150	32.19±3.22	13.33±0.43
	Alg on 3D Ti23	0.151±0.180	0.668±0.169	0.404±0.076	50.20±1.05	2.15±0.15
	Alg/OCP on 3D Ti23	0.170±0.041	0.803±0.053	0.550±0.149	25.41±2.99	5.51±0.24

Supplementary Table S2. Parameters determined from fitting of the EIS plots of the hydrogel coatings on 3D-printed Ti Gr2 and Ti Gr23 substrates.

Simulated conditions	Specimens	R_{ct} (kΩ·cm^2)	Q_{dl} ($10^{-5}Ω^{-1}·cm^{-2}·S^n$)	n_1	R_c (kΩ·cm^2)	Q_c ($10^{-5}Ω^{-1}·cm^{-2}·S^n$)	n_2
Normal	Alg on 3D Ti2	17.63±0.33	1.16±0.29	0.89	12.33±0.83	1.05±0.16	0.85
	Alg/OCP on 3D Ti2	23.09±0.10	1.04±0.12	0.92	18.84±0.75	1.02±0.14	0.86
	Alg on 3D Ti23	20.66±0.73	0.84±0.15	0.88	16.07±0.26	1.04±0.37	0.92
	Alg/OCP on 3D Ti23	25.12±0.39	0.69±0.05	0.88	21.30±0.79	0.96±0.08	0.94
Inflammatory	Alg on 3D Ti2	8.15±0.02	1.24±0.21	0.95	5.40±0.60	1.19±0.39	0.87
	Alg/OCP on 3D Ti2	9.86±0.80	1.66±0.19	0.93	7.15±0.91	1.50±0.50	0.96
	Alg on 3D Ti23	10.20±0.54	1.32±0.17	0.90	9.06±0.27	1.22±0.36	0.83
	Alg/OCP on 3D Ti23	11.54±0.04	1.57±0.62	0.95	9.28±0.57	1.16±0.20	0.90
Severe inflammatory	Alg on 3D Ti2	3.10±0.13	1.43±0.20	0.91	2.53±0.65	1.06±0.54	0.94
	Alg/OCP on 3D Ti2	4.59±0.58	1.87±0.23	0.89	3.01±0.53	1.73±0.17	0.89
	Alg on 3D Ti23	3.12±0.80	2.09±0.39	0.89	2.75±0.77	1.24±0.51	0.90
	Alg/OCP on 3D Ti23	4.85±0.95	1.65±0.64	0.92	4.09±0.12	1.13±0.28	0.89
